# Diagnostic accuracy of Dual-Energy CT in detecting traumatic vertebral bone marrow edema: a prospective comparative study with MRI in the context of a level I trauma center

**DOI:** 10.1007/s00234-025-03763-2

**Published:** 2025-09-22

**Authors:** Thomas Beyer, Erik Volmer, Patrick Gahr, Marc-André Weber

**Affiliations:** 1 Institute of Diagnostic and Interventional Radiology, Paediatric Radiology and Neuroradiology, Rostock University Medical Center, Rostock, Germany; 2 Department of Trauma, Hand and Reconstructive Surgery, Rostock University Medical Center, Rostock, Germany

**Keywords:** Dual-Energy CT, Bone marrow edema, Vertebral fracture, Trauma, Diagnostic accuracy, Cost-effectiveness

## Abstract

**Background:**

Traumatic vertebral fractures present a significant diagnostic challenge in emergency settings. Magnetic resonance imaging (MRI) excels in detecting bone marrow edema but faces practical limitations in acute trauma care. This prospective study evaluates the diagnostic accuracy of Dual-Energy Computed Tomography (DECT) in detecting traumatic vertebral bone marrow edema within a Level I trauma center environment.

**Methods:**

Between May 2020 and July 2023, 291 DECT examinations were performed on adult patients presenting with suspected or confirmed spinal injury. From these, 233 (80.1%) met quality criteria for analysis. A subgroup of 47 patients underwent additional MRI as reference standard, with 44 (93.6%) providing diagnostically evaluable images. Two board-certified radiologists independently assessed vertebral bone marrow edema presence in blinded, randomized evaluations using both modalities. Diagnostic parameters, examination times, radiation exposure, and cost-efficiency were analyzed.

**Results:**

DECT demonstrated an overall sensitivity of 82.9% and specificity of 96.6% for detecting vertebral bone marrow edema compared to MRI. The thoracolumbar junction showed highest sensitivity (91.7% for L3). DECT examination time was 7.2 minutes (including post-processing) versus 12 minutes for MRI, meaning MRI required 66.7% more time than DECT. DECT radiation exposure showed a mean dose-length product increase of only 3% compared to conventional CT. Body mass index showed no significant influence on DECT interpretability (p=0.196) or diagnostic accuracy except in isolated segments (L3, T11). Cost-benefit analysis revealed potential savings of 49.1% (€104.40) per spinal segment with DECT-based diagnostic pathways.

**Conclusion:**

DECT offers high diagnostic accuracy for detecting traumatic vertebral bone marrow edema with substantial time and cost advantages compared to MRI. The technique demonstrates particular value in acute trauma settings, while acknowledging limitations from artifacts (19.9% of cases) primarily caused by medical devices. These findings support implementing DECT as an efficient alternative to MRI in spinal trauma diagnostics.

## Introduction

In trauma and emergency medicine, spinal trauma—particularly vertebral fractures—represents a common and prognostically significant entity in multiply injured patients. Computed tomography (CT) has become the first-line modality for initial evaluation due to its high availability, speed, and excellent spatial resolution [1]. However, the assessment of trabecular bone, which is primarily affected in vertebral fractures, remains diagnostically challenging. Due to its lower mechanical strength compared to cortical bone, the trabecular compartment is particularly susceptible to deformation and collapse. Accurate evaluation is therefore essential to identify acute fractures and distinguish them from chronic or incidental findings.

Acute injuries often manifest as architectural disruption of trabeculae, accompanied by hemorrhagic infiltration and interstitial edema. In Dual-Energy CT (DECT), particularly on virtual non-calcium (VNCa) images, these changes appear as areas of increased attenuation due to selective calcium suppression and elevated water content. This allows indirect visualization of bone marrow edema (BME), a surrogate marker for fracture acuity. While CT can only depict such changes indirectly, magnetic resonance imaging (MRI) with fat-suppressed sequences offers superior sensitivity for detecting and characterizing BME [2–4].

The implementation of MRI in acute trauma care is often limited by prolonged acquisition times, limited availability, contraindications (e.g., pacemakers), and the need for sedation [5]. Moreover, MRI is primarily indicated for assessing the spinal cord, neural elements, and posterior ligamentous complex. The detection of BME, although not a standalone indication, remains a valuable adjunct in assessing fracture acuity and guiding treatment decisions.

DECT expands the diagnostic capabilities of CT by utilizing spectral imaging with two X-ray energy levels (typically 80 and 140 kV), enabling material decomposition based on the energy-dependent attenuation of different tissues. VNCa images derived from DECT suppress the calcium signal in bone, allowing edematous marrow to appear hyperdense compared to surrounding bone. This technique provides an indirect surrogate for BME detection [6–9]. While previous studies have already demonstrated high diagnostic agreement between DECT and MRI in acute vertebral fractures [9–12], this study evaluates the method in the setting of a level I trauma center with a heterogeneous patient population. Specifically, we assess the diagnostic performance of DECT for detecting vertebral BME using MRI as the reference standard.

In addition, this study examines the influence of body mass index (BMI) on DECT image interpretability and diagnostic accuracy—a factor not systematically addressed in the current literature. Furthermore, we perform a matched dosimetric comparison with conventional CT, evaluate examination times including post-processing, and conduct a cost-benefit analysis based on current reimbursement standards.

## Materials and methods

### Objectives and study design

This prospective study evaluates the diagnostic accuracy of Dual-Energy Computed Tomography (DECT) in detecting acute vertebral fractures and associated bone marrow edema in the context of emergency medicine. Non-contrast Magnetic Resonance Imaging (MRI) with edema-sensitive, fat-saturated T2-weighted sequences serves as the reference standard. The primary research objectives address the diagnostic potential of DECT as an equivalent to MRI and its implications for process optimization and cost efficiency in acute and intensive care settings.

### Study protocol and patient cohort

The study was conducted in two phases:

I. Initial evaluation and protocol optimization of DECT in the clinical emergency setting.
II. Main phase comprising 291 DECT examinations, of which 233 (80.1%) met the required quality criteria. In 47 patients of this cohort, additional MRI was performed as a reference standard, with 44 (93.6%) MRI examinations being diagnostically evaluable.

The study consecutively included all adult patients (≥ 18 years) who presented to the trauma bay or central emergency department for acute diagnostic imaging due to clinically suspected or confirmed spinal injury. The examinations were conducted between May 4, 2020, and July 27, 2023. Inclusion required the absence of contraindications for the necessary CT or MRI examination. Exclusion criteria comprised (1) patients under 18 years of age (*n* = 3) and (2) cases in which the initial clinical suspicion of spinal injury was not supported by further physical examination or preliminary imaging, thereby not warranting acute spinal imaging (*n* = 9). No additional exclusion criteria such as contraindications to imaging or lack of clinical data applied. All included patients were eligible for both CT and, where applicable, MRI according to clinical indication. The final patient cohort (*n* = 233) had a mean age of 74.7 years, with an age range of 19 to 102 years and a gender distribution of 56.7% female to 43.3% male patients.

### Imaging modalities

CT diagnostics were performed using two CT systems (256-detector rows, Revolution™, GE HealthCare), equipped with Gemstone Spectral Imaging (GSI) technology for dual-energy scanning. This technology enabled simultaneous acquisition at two different energy levels (80 kV and 140 kV), allowing differentiated material characterization. MRI examinations were conducted on a 1.5 T SIGNA™ Artist (*n* = 4) and two identical 3.0 T SIGNA™ Premier systems (*n* = 43) (GE HealthCare).

### Image analysis

The analysis of DECT datasets, including the generation of color-coded edema maps, was performed using dedicated post-processing software (AW-Server, Version 3.2, GE HealthCare). Two board-certified radiologists (with 9 and 6 years of experience in musculoskeletal imaging) independently conducted qualitative image analysis. Assessment was performed qualitatively. The DECT and MRI datasets were evaluated in a blinded and randomized manner on the same day. Each recorded vertebral body was evaluated in both modalities for the presence of bone marrow edema. The assessment was dichotomous (edema present/absent) and was documented separately for each modality.

Statistical Analysis: Statistical evaluation was performed using IBM™ SPSS™ Statistics (Version 27, IBM Corp., Armonk NY, USA). Diagnostic parameters such as sensitivity, specificity, positive and negative predictive values were calculated. The interreader reliability was assessed using Cohen’s Kappa coefficient, which yielded a value of 0.94, indicating almost perfect agreement between the two radiologists. Kappa values were interpreted based on the guidelines by Landis and Koch [13]. A significance level of 0.05 was applied for all statistical tests. Logistic regression analysis was performed to assess the influence of body mass index (BMI) on the interpretability of DECT and MRI. The omnibus test of model coefficients was used to evaluate the significance of BMI as a predictor variable. The statistical analysis was conducted by one of the two radiologists (readers) involved in the image evaluation. This approach ensured consistency in interpreting the dataset, with potential bias minimized through strict blinding and randomization of the imaging data.

### Dosimetric analysis

For evaluation of radiation dose, DECT examinations were compared with conventional CT examinations. For this purpose, a matched comparison cohort was formed for each age decade, consisting of three randomly selected patients with monoenergetic CT imaging per decade. Matching criteria included gender, BMI, and the examined spinal segment. The dose-length product (DLP) served as the comparison parameter.

### Analysis of examination times

Recording of examination times was performed for both DECT and MRI protocols. For DECT, in addition to the pure scanning time, the time required for post-processing using dedicated edema mapping software was documented. For MRI, the total sequence runtime was recorded. The time measurement took into account individual adaptations of examination protocols to patient size and examination region.

### Cost-benefit analysis

A cost-benefit analysis was performed to evaluate the financial impact of performing initial DECT examinations compared to the conventional diagnostic pathway of standard CT followed by MRI. The costs were calculated based on current reimbursement rates according to the German Uniform Evaluation Standard (Einheitlicher Bewertungsmaßstab, EBM), with €82.04 per spinal segment for CT/DECT (procedure code 34311) and €130.50 per spinal segment for MRI (procedure code 34411). The analysis considered both direct examination costs and the diagnostic pathway efficiency. To determine the potential cost savings, we established the proportion of cases where DECT provided sufficient diagnostic information, thereby eliminating the need for subsequent MRI. The potential cost reduction was calculated by comparing the costs of two diagnostic pathways: (1) initial DECT without subsequent MRI in cases with sufficient diagnostic certainty, and (2) conventional CT followed by MRI in all cases. This analysis takes into account the proportion of diagnostically interpretable DECT examinations (80.1% as observed in this study) and the high interreader reliability (κ = 0.94), indicating diagnostic confidence that would obviate the need for additional MRI examinations.

## Results

### Radiation dose

The comparative analysis of dose-length products (DLP) showed that DECT examinations had a mean value 3% higher than monoenergetic CT examinations (range: −8% to + 12%). The variability in DLP values primarily correlated with the craniocaudal extent of the examined spinal segment. Across all DECT examinations, the minimum DLP was 193.0 mGy*cm, the maximum was 602.3 mGy*cm, with a mean of 336.5 mGy*cm and a standard deviation of 113.0 mGy*cm.

### Examination times

The average protocol duration for DECT examination including scout was 13 s, with an additional post-processing time of 7 min for generating edema color maps (total: 7.2 min). MRI protocols required an average scan time of 12 min. The interpretation time did not differ significantly between the two modalities. Thus, MRI examinations required 66.7% more time compared to DECT including post-processing. Protocol times for both modalities varied depending on the anatomical extent of the examination region and patient size.

**Table:** 

Criterion	DECT	MRI
Scan time (including scout)	13 s	12 min
Post-processing	7 min	–
Total duration	7 min 13 s	12 min
Interpretation time	No sign. difference	No sign. difference

### Number and quality of DECT examination interpretability by region

**Fig. 1:**
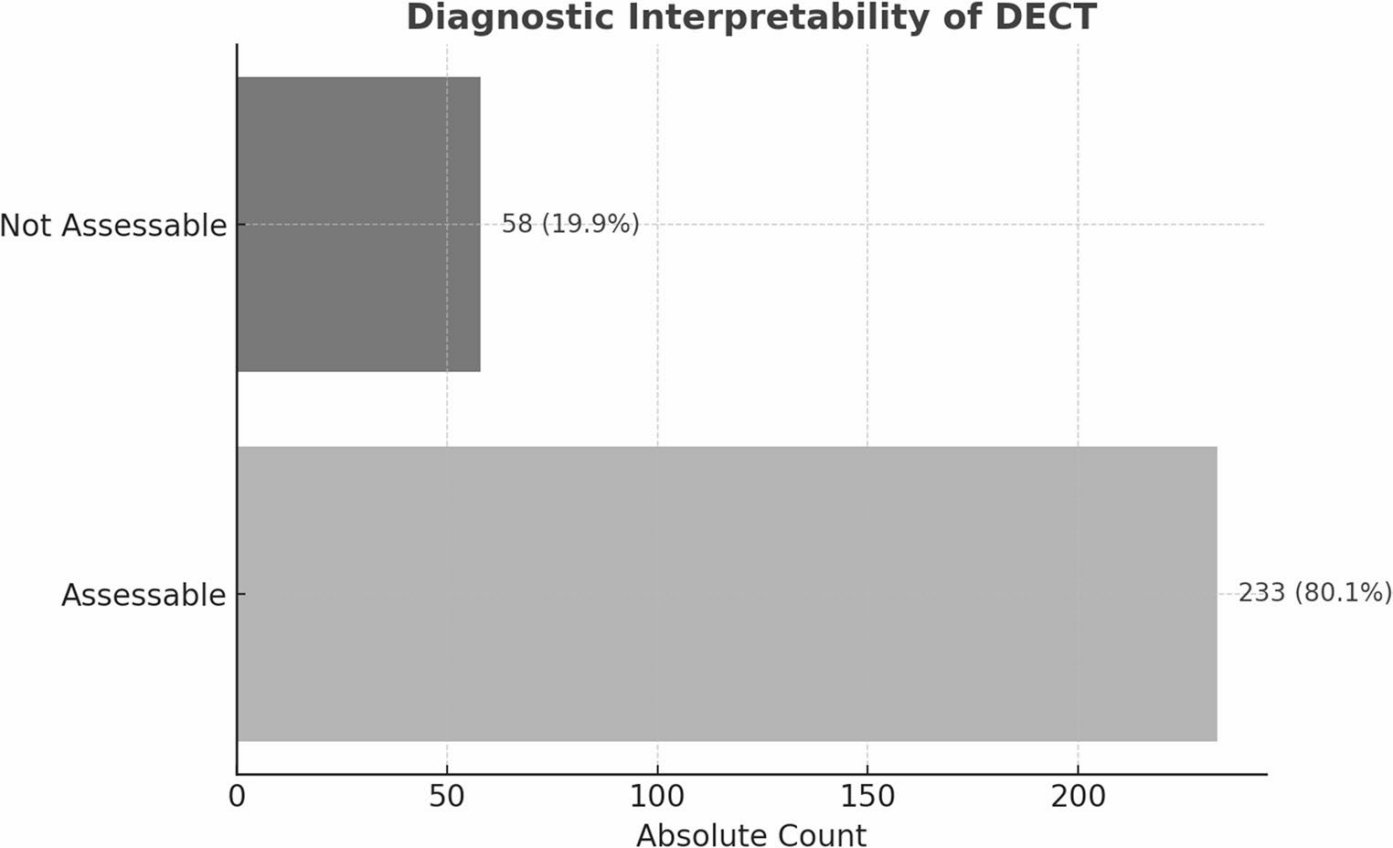
Diagnostic interpretability of DECT in the study cohort (*n* = 291). A total of 233 examinations (80.1%) were deemed diagnostically assessable based on post-processing quality and image readability. In 58 cases (19.9%), image evaluation was not feasible due to artifacts or insufficient diagnostic quality

Figure 1 shows the absolute number of examinations and their proportion of diagnostically interpretable examinations.

Overall, 233 out of 291 DECTs were diagnostically interpretable. The vast majority (97.4%) of identified vertebral bone marrow edema was detected in the thoracic and lumbar segments.

Despite the relatively low number (*n* = 6) of cervical vertebral edema, a comparable rate of diagnostically significant DECT examinations was achieved in this region, with this rate being at least 80% of the performed examinations.

### Sensitivity and specificity of DECT

**Table:** 

Vertebral body	Number of edemas in DECT	Percentage of all edemas (%)	Sensitivity (%)	Specificity (%)
**C1**	0	0	-	-
**C2**	0	0	-	-
**C3**	1	0.6	100	100
**C4**	1	0.6	100	100
**C5**	0	0	-	-
**C6**	2	1.3	50	100
**C7**	2	1.3	100	100
**T1**	1	0.6	-	-
**T2**	4	2.5	100	100
**T3**	13	8.2	50	100
**T4**	10	6.3	0	100
**T5**	15	9.4	66.7	100
**T6**	12	7.5	50	100
**T7**	12	7.5	100	100
**T8**	16	10.1	80	100
**T9**	15	9.4	60	100
**T10**	20	12.6	60	100
**T11**	26	16.4	66.7	90
**T12**	32	20.1	63.6	100
**L1**	59	37.1	82.4	100
**L2**	33	20.8	71.4	85.7
**L3**	25	15.7	91.7	91.7
**L4**	13	8.2	40	60
**L5**	8	5.0	50	75

Overall Sensitivity: 82.9%

Overall Specificity: 96.6%

The segment-specific analysis of DECT’s diagnostic performance demonstrated an overall sensitivity of 82.9% and an overall specificity of 96.6%. Individual analysis of vertebral segments showed that 17 out of 19 evaluable vertebral bodies demonstrated specificity ≥ 85%, while sensitivity exhibited segment-dependent variations. The highest sensitivity values were achieved in the thoracolumbar junction, with peak values of 91.7% for L3.

The diagnostic performance of DECT in detecting bone marrow edema is exemplarily illustrated in Appendix Figs. 2 and 3. Appendix Fig. 2 presents the case of a 60-year-old male patient after multiple falls from 2 to 3 m height with an acute vertebral fracture of L1 and a subacute burst fracture of L3. Appendix Fig. 3 demonstrates the DECT and MRI findings of an 84-year-old female patient after falling from a nursing bed with an acute T12 fracture and an endplate fracture of L1. Additionally, edema-equivalent bone marrow changes are detectable in T2-4. In both cases, there is concordance between DECT and simultaneously performed non-contrast MRI, independent of age, gender, and the presence of degenerative changes.

### Impact of BMI on the interpretability and diagnostic accuracy of DECT and MRI

The results of logistic regression analysis demonstrate that BMI does not significantly influence the interpretability of DECT, as the omnibus test of model coefficients is not significant (*p* = 0.196). Similarly, analysis for MRI revealed that BMI has no significant impact on its interpretability (*p* = 0.961). These findings suggest that both modalities can provide adequate diagnostic information regardless of the patient’s height or body mass.

### Influence of BMI on diagnostic sensitivity and specificity of DECT

Regarding the diagnostic accuracy of DECT, a differentiated picture emerges. While BMI had no significant influence on DECT sensitivity for most vertebral bodies, the analysis revealed a significant impact for vertebral body L3 (*p* = 0.026). The regression model explained approximately 59.1% of sensitivity variance, indicating a strong relationship between BMI and DECT sensitivity in this region. However, no significant correlation was found for other vertebral bodies, suggesting that BMI influences DECT’s diagnostic sensitivity only in exceptional cases.

The specificity of DECT also showed heterogeneous results. For most vertebral bodies, BMI was not significant; however, a significantly strong influence of BMI on DECT specificity was demonstrable for both vertebral body L3 (*p* = 0.026) and T11 (*p* = 0.031). In these regions, BMI explained 59.1% and 72.6% of specificity variance, respectively, indicating a substantial relationship between patient BMI and DECT diagnostic precision in these segments. For the remaining vertebral bodies, no significant relationship between BMI and DECT specificity could be established.

### Cost-benefit analysis results

The cost comparison between diagnostic pathways revealed significant potential savings with the DECT-based approach. In the conventional pathway, the combined cost for CT followed by MRI was €212.54 per spinal segment (€82.04 + €130.50). In contrast, the cost of DECT alone was €82.04 per spinal segment. Based on our findings that 80.1% of DECT examinations were diagnostically interpretable with high specificity (96.6%) and acceptable sensitivity (82.9%), we estimated that in these cases, no subsequent MRI would be required, resulting in a cost of €82.04 per segment. For the remaining 19.9% of cases where DECT provided insufficient diagnostic information, additional MRI would be necessary, resulting in a combined cost of €212.54 per segment.

The weighted average cost per patient for the DECT-based pathway was therefore €108.14 per spinal segment (€82.04 × 0.801 + €212.54 × 0.199), representing a potential cost reduction of €104.40 (49.1%) per spinal segment compared to the conventional pathway. This significant per-segment cost reduction demonstrates the economic potential of implementing DECT as the primary diagnostic modality in spinal trauma evaluation.

## Discussion

The comparative analysis of examination modalities demonstrates significant differences regarding required examination times. DECT enables considerably more time-efficient diagnostics with a total examination time of 7.2 min (13 s scan time plus 7 min post-processing) compared to MRI (12 min, corresponding to an additional time expenditure of 66.7%). The radiation exposure of DECT, with a mean DLP increase of 3% (range: −8% to + 12%), is only slightly above that of conventional CT examinations. All DLPs were within the diagnostic reference levels established by the Federal Office for Radiation Protection (Bundesamt für Strahlenschutz), when normalized to the respective scan lengths (DLP = CTDIvol × scan length).

Limited diagnostic utility of DECT was found in 58 cases (19.9%) due to restricted interpretability, primarily attributable to artifacts from medical devices (43 cases, 14.8%), followed by motion artifacts in agitated or pain-afflicted patients (15 cases, 5.1%). These limitations need to be considered when implementing DECT in clinical workflow, particularly in intensive care settings [14, 15].

The cost-benefit analysis demonstrates substantial financial advantages of implementing DECT as the primary diagnostic modality in spinal trauma. The potential cost reduction of 49.1% per spinal segment (€104.40) represents a significant financial benefit while maintaining diagnostic quality. This finding aligns with the economic evaluation by Wong et al. [16], who reported similar cost efficiency of DECT in trauma diagnostics by demonstrating a significant reduction in follow-up MRI utilization and associated healthcare expenditures. The economic advantage becomes particularly relevant in healthcare systems with limited resources and high patient volumes, such as level I trauma centers. Furthermore, the cost efficiency must be considered in conjunction with the substantial time savings of 66.7% compared to MRI, which has additional economic implications through improved resource allocation and accelerated patient management. The combination of economic benefits with the high diagnostic reliability of DECT provides a compelling argument for its implementation in clinical routine. However, it must be noted that our cost analysis was based on the current reimbursement rates according to the German Uniform Evaluation Standard (EBM), which may vary between healthcare systems and over time. Additionally, the analysis did not account for secondary economic factors such as reduced hospitalization duration due to faster diagnostics or the economic impact of potential diagnostic errors. These aspects should be addressed in future comprehensive health economic evaluations.

The results of the present study regarding the diagnostic accuracy of DECT in spinal trauma demonstrate high concordance with the findings of a recent meta-analysis on vertebral fractures by Ghazi Sherbaf et al. [9] (pooled sensitivity 89%, specificity 96%), showing an overall sensitivity of 82.9% and an overall specificity of 96.6%. In particular, the high specificity confirms the method’s reliability in excluding bone marrow edema. The slightly lower sensitivity in this study compared to published data might be attributable to the heterogeneous distribution of case numbers across different spinal segments. While this heterogeneity reflects the real distribution of spinal injuries in clinical practice, it limits statistical significance in less frequently affected segments. However, it should be noted that the comparability of results between different studies may be limited due to the use of different DECT technologies. While Gemstone Spectral Imaging (GSI) and Layered Detector techniques typically provide higher spectral resolution that improves material differentiation, fast kVp switching offers advantages in reducing motion artifacts, which could impact diagnostic accuracy.

In this context, emerging detector technologies such as photon-counting CT (PCCT) represent a promising future direction in spectral imaging. PCCT provides higher spatial resolution, improved contrast-to-noise ratios, and more refined material decomposition compared to conventional energy-integrating detectors. Especially in musculoskeletal radiology, where detailed visualization of trabecular microarchitecture and extended anatomical coverage are critical, PCCT has shown considerable potential to enhance diagnostic performance [17]. Although its clinical use in trauma imaging is still limited, it may offer particular advantages in challenging anatomical regions or in patients with metallic implants. Incorporating DECT into this evolving technological landscape underscores its current clinical value, while future research should explore whether photon-counting systems can further improve fracture characterization and edema detection.

Further validation of DECT’s diagnostic performance requires prospective studies with larger case numbers, particularly in less frequently affected spinal segments. Of particular interest would be the evaluation of reader expertise [18] and systematic analysis of potential factors contributing to false-negative findings.

Beyond methodological performance, the clinical relevance of detecting bone marrow edema (BME) lies in its role as a surrogate marker for fracture acuity. BME reflects trabecular microarchitectural disruption and interstitial hemorrhage and is therefore critical for distinguishing acute from chronic or incidental vertebral deformities. This distinction is particularly important in elderly or osteoporotic patients, where vertebral height loss is common and BME may be the only indicator of a recent, potentially unstable fracture. Identifying such edema informs therapeutic decisions—such as the need for surgical stabilization, bracing, or conservative management—and enhances diagnostic confidence when cortical disruption is subtle or absent.

The logistic regression analysis of body mass index (BMI) revealed no significant correlation with the diagnostic interpretability of DECT (*p* = 0.196) or MRI (*p* = 0.961). In the segment-specific analysis, a significant BMI influence on DECT’s diagnostic accuracy was observed only in segments L3 and T11. However, these isolated findings do not support the inference of a systematic BMI-dependent relationship with diagnostic parameters. Thus, based on the available data, the hypothesis that BMI as a singular factor could determine the choice of modality between DECT and MRI cannot be confirmed.

Overall, DECT appears to be a promising alternative to MRI in acute diagnostics for patients with spinal trauma. The significantly shorter examination times with comparable diagnostic accuracy to MRI offer additional advantages in clinical practice. Further optimization of examination protocols and standardized reader training could further enhance diagnostic performance.

Compared to previous publications on DECT in vertebral trauma, our study offers several novel contributions. It is one of the largest prospective single-center cohorts evaluating DECT in a level I trauma setting. It includes a segment-level analysis of diagnostic accuracy across the entire spine and is, to our knowledge, the first to systematically assess the influence of body mass index (BMI) on DECT sensitivity and specificity. Additionally, the study provides a matched dosimetric comparison with conventional CT, a time-efficiency analysis including post-processing, and a cost-benefit evaluation based on current German EBM reimbursement rates. These aspects have not been comprehensively addressed in earlier studies and underline the added clinical and methodological value of our findings.

### Limitations

A limiting factor in the present cohort was the occurrence of artifacts affecting image quality, particularly in patients with extracorporeal devices such as ECG electrodes, ventilation, and blood pressure equipment. Due to their high X-ray density, these devices frequently induce multiple artifacts and attenuation differences that significantly complicate both image quality and subsequent evaluation. Such artifacts can sometimes completely preclude the assessment of adjacent vertebral body segments regarding the presence of bone marrow edema, further limiting diagnostic reliability in certain cases.

Appendix Fig. 4 shows a DECT of the thoracolumbar junction in sagittal reconstructions of a trauma patient following thoracic injury, who, in conjunction with previous imaging not presented here, demonstrates a new endplate impression fracture of T9 and a new burst fracture of T11. In the color-coded edema-sensitive post-processing images, extensive bone marrow edema is detected in T5-10. There is no evidence of edema-equivalent signal changes in T11. Additionally visible is a cardiac pacemaker with an outgoing lead overlying the described vertebral bodies T5-11. Therefore, artifact-induced signal changes must be considered, which may merely imitate the presence of bone marrow edema in the described vertebral bodies.

In another example, Appendix Fig. 5 demonstrates insufficient edema detection by DECT compared to fat-suppressed T2 imaging of a non-contrast MRI due to severe artifacts that cannot be eliminated. This again emphasizes that the examined vertebral region has a significant influence on sensitivity and specificity, at least in the examined cervical spine region.

### Summary

The present study evaluates the diagnostic performance of Dual-Energy Computed Tomography (DECT) in detecting bone marrow edema in spinal trauma in a level I trauma center. DECT demonstrated high diagnostic accuracy with an overall sensitivity of 82.9% and specificity of 96.6%, comparable to published meta-analysis data. The method proved time-efficient with a total examination time of 7.2 min compared to MRI (12 min), with only slightly increased radiation exposure (mean DLP increase: 3%) compared to conventional CT.

Cost-benefit analysis revealed a potential cost reduction of 49.1% per spinal segment with DECT as the primary diagnostic tool compared to the conventional CT plus MRI pathway.

Diagnostic utility was impaired in 19.9% of cases by artifacts, primarily caused by medical devices (14.8%) and motion artifacts (5.1%). Body Mass Index showed no significant influence on diagnostic interpretability for either modality, except for isolated segment-specific effects in L3 and T11.

These findings support the use of DECT as an efficient alternative to MRI in acute diagnosis of spinal trauma, while considering method-specific limitations, particularly in intensive care settings.

## Figures and Tables

**Fig. 2:**
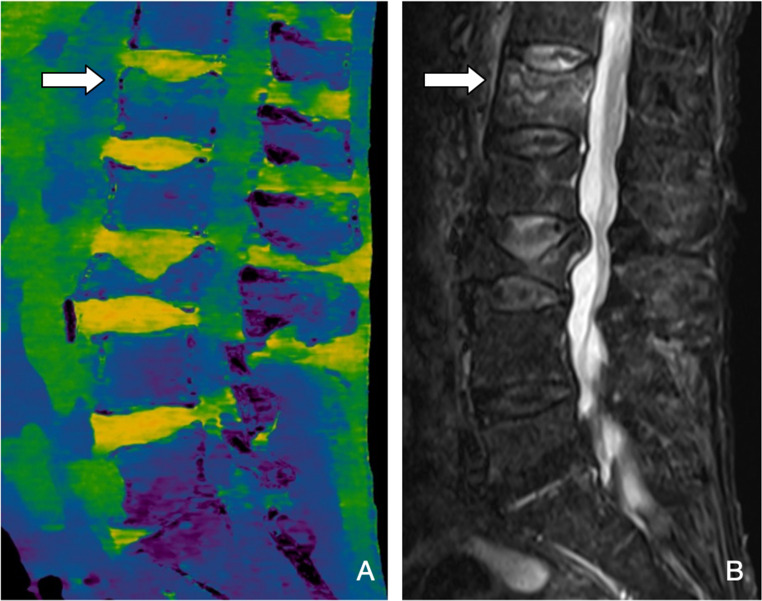
**A** Color-coded map from DECT in sagittal reconstruction. Edema-equivalent signal alterations are seen in L1 (arrow). The height-reduced L3 shows only minimal signal changes, consistent with an older osteoporotic compression fracture with residual marrow changes. **B** Corresponding fat-suppressed sagittal T2-weighted MIR (STIR) demonstrates matching findings with a marked signal increase in L1 and only faint signal in L3, supporting the impression of a chronic fracture

**Fig. 3:**
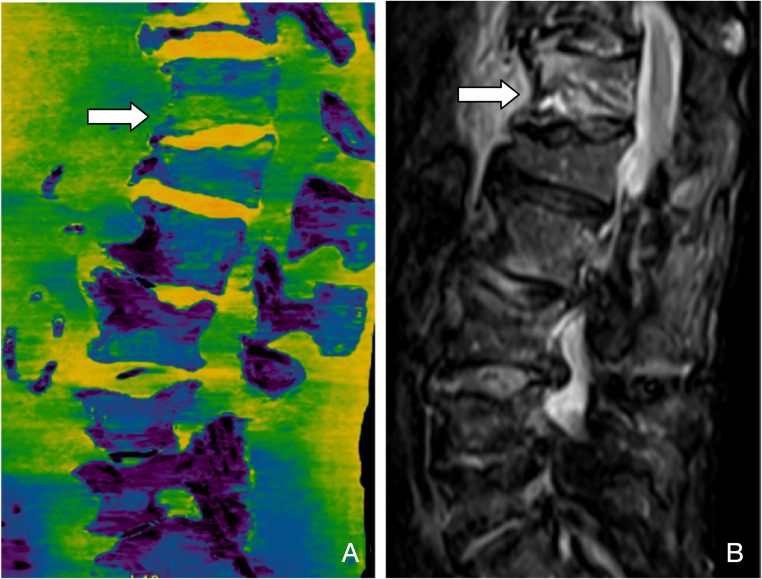
**A** Color-coded map from DECT in sagittal reconstruction; pronounced edema-equivalent signal alternations in the inferior endplate of T12 (arrow) and the superior endplate of L1, as well as mild diffuse involvement of L2-4. **B** Fat-suppressed sagittal T2-weighted MRI (STIR) showing corresponding edema-equivalent signal increases in the described vertebral bodies

**Fig. 4:**
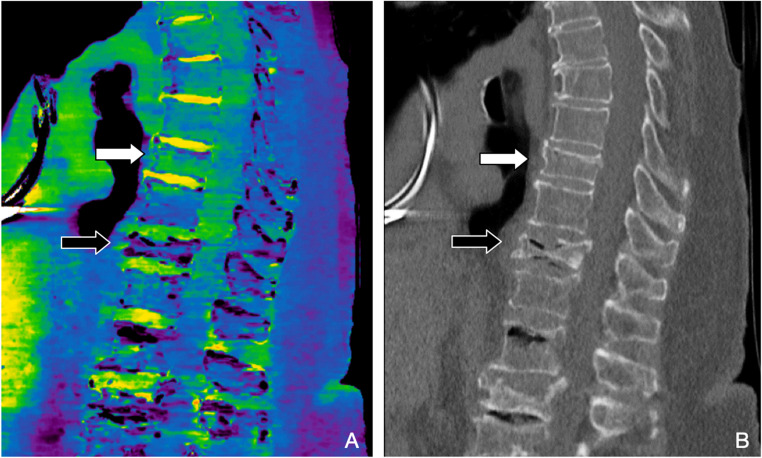
Patient after thoracic trauma. **A** Color-coded map from DECT showing endplate impression fracture of T9 (white arrow). The vertebral body of T11 (black arrow) shows no edema-equivalent signal, despite structural deformity, consistent with a chronic osteoporotic burst fracture. **B** Sagittal reconstruction of the CT bone kernel

**Fig. 5:**
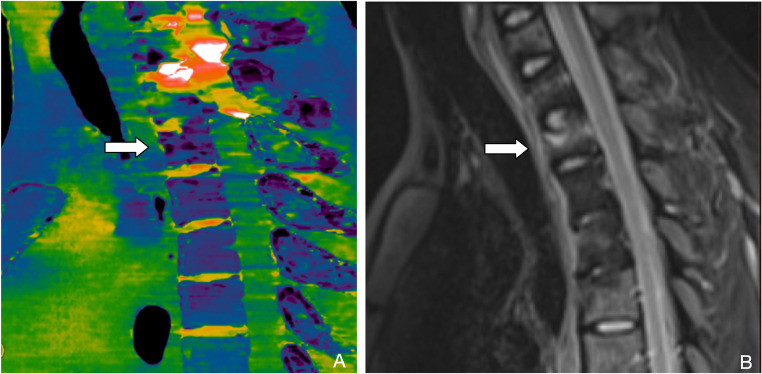
**A** Color-coded map from DECT in sagittal reconstruction; pronounced artifacts masking the bone marrow edema of T1 (arrow) that is distinguishable in MRI imaging. **B** Fat-suppressed sagittal T2-weighted MRI (STIR) showing corresponding edema-equivalent signal increases in T1. Also showing minor involvement of T4 and T5

**Fig. 6:**
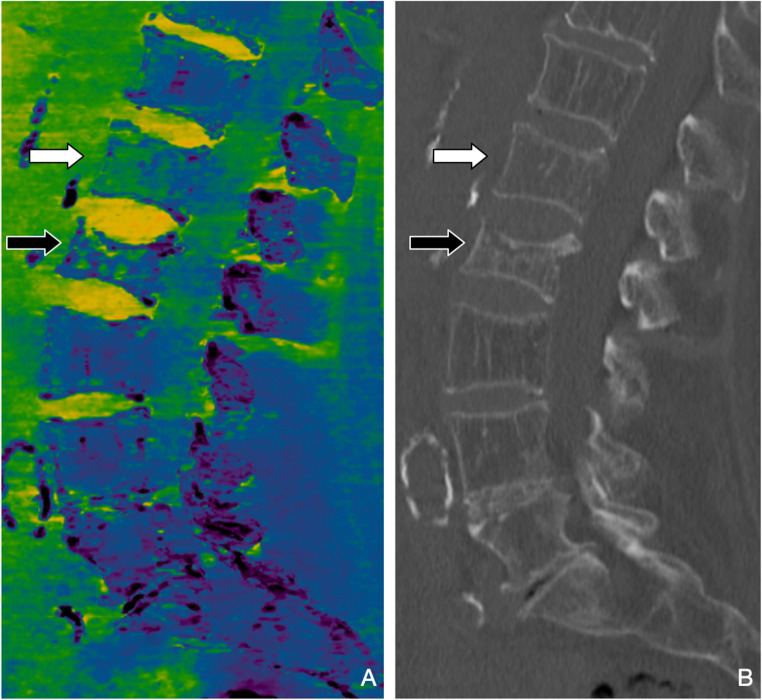
97-year-old woman with atraumatic lumbar spine and pelvic pain. **A** Pronounced edema-equivalent signal alterations of the bone marrow at L1 (white arrow) on the color-coded map from DECT with only minimal height reduction of the vertebral body. Distinct endplate collapse with patchy edema-equivalent signal alterations of the bone marrow at L2 (black arrow). **B** Sagittal CT reconstruction with bone kernel. Posterior protrusions of both fractures into the spinal canal are visible. Overall pronounced rarefaction of trabecular bone structure in the setting of severe osteoporosis

**Fig. 7:**
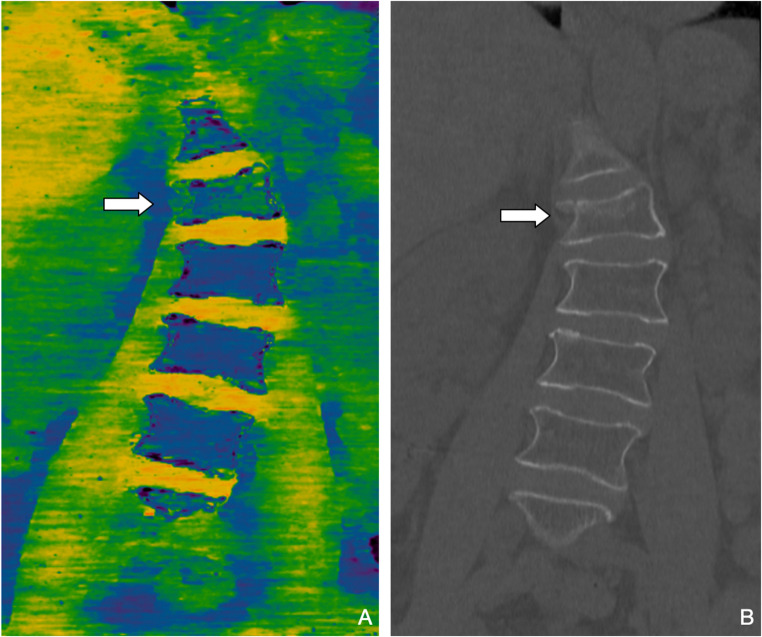
67-year-old woman with stair fall on the day of examination*.***A **Coronal view of the lumbar spine showing diffuse edema-equivalent signal alterations at L1 (white arrow) on the color-coded map from DECT.**B **Coronal CT reconstruction with bone kernel. A pronounced left convex scoliosis of the lumbar spine is evident

## Data Availability

No datasets were generated or analysed during the current study.

## Appendix

See Figs. 2, 3, 4, 5, 6 and 7
